# Superior Vena Cava Syndrome from Pacemaker Leads in an Elderly Woman

**DOI:** 10.7759/cureus.40323

**Published:** 2023-06-12

**Authors:** Michael C Witte, Ron Garry

**Affiliations:** 1 Graduate Medical Education, North Collier Hospital (NCH) Healthcare System, Naples, USA; 2 Geriatrics, North Collier Hospital (NCH) Healthcare System, Naples, USA

**Keywords:** device-related thrombus (drt), superior vena cava thrombosis, geriatric medicine, pacemaker complication, superior vena cava (svc) syndrome

## Abstract

Superior vena cava (SVC) syndrome is a constellation of clinical signs and symptoms secondary to obstruction of the SVC. The most common etiology is malignancy. However, the incidence of device-related SVC syndrome is increasing. The current management for device-related SVC syndrome includes open surgical intervention, endovascular repair, or systemic anticoagulation. We present a case of a 95-year-old female who developed SVC syndrome due to thrombosis secondary to pacemaker leads. She was treated conservatively due to her frailty and comorbid conditions. We discuss the evolving etiology of SVC syndrome, the most common presenting signs and symptoms, and a variety of potential treatments for non-malignant SVC syndrome. We also provide an example of when conservative management for chronic device-related SVC syndrome is appropriate.

## Introduction

Superior vena cava (SVC) syndrome is caused by obstruction of the SVC. The SVC is formed by the junction of the right and left brachiocephalic veins with additional major tributaries being the internal thoracic vein and the azygos vein. It is the primary venous drainage system of the upper extremities, face, and neck. Obstruction of the SVC causes a group of signs and symptoms that is termed “superior vena cava syndrome.” The most common etiology of SVC obstruction is malignancy, which comprises approximately 60-65% of cases [1,2]. The other 35-40% of cases are from benign causes; indwelling devices are the most common etiology in this group [1,2]. The clinical signs and symptoms, along with imaging, elucidate the diagnosis. Treatment of the obstruction depends on the etiology, but current options include radiation, chemotherapy, thrombolysis, open surgical intervention, angioplasty, stenting, and systemic anticoagulation.

## Case presentation

**Figure 1:**
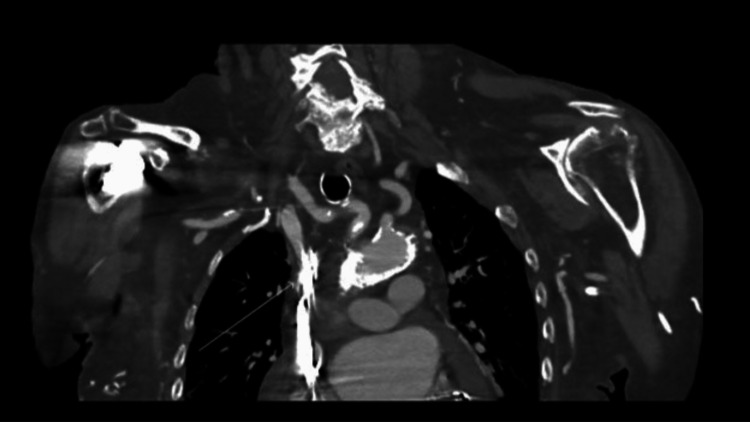
Computed tomography with intravenous contrast of the chest in the coronal plane showing the point of superior vena cava obstruction and a conglomeration of functional and abandoned pacemaker wires (white arrow).

A 95-year-old female with a history significant for dual-chamber permanent pacemaker implantation secondary to complete AV-node block, coronary artery disease, heart failure with preserved ejection fraction, paroxysmal atrial fibrillation, hypertension, hypercholesteremia, gastrointestinal bleeding, gastroesophageal reflux disease, and microscopic colitis presented to the primary care clinic with edema of the face, neck, and clavicles that had progressed over the past three months. The patient originally had a right ventricular permanent pacemaker placed in 1993 that was upgraded to a dual-chamber pacemaker in 2016. Upon presentation, the patient denied shortness of breath, dyspnea on exertion, pain, cough, or hoarseness. Physical exam was notable for facial edema, clavicular fullness, and jugular venous distention. Cyanosis and upper neck and chest venous congestion were absent. Initial differential diagnosis included Cushing’s syndrome secondary to budesonide treatment for her microscopic colitis. However, there was no improvement in the patient’s symptoms after withdrawing the medication. Computed tomography of the chest with intravenous contrast was ordered to evaluate for SVC syndrome. The results showed chronic central venous occlusion with an extensive network of well-established collateral veins providing venous return to the right atrium (Figure 1). Complete blood count was notable for a white blood cell count of 6.3 cells/mcL, hemoglobin of 11 g/dL, hematocrit of 36%, mean corpuscular volume of 105.2 fL, and platelet count of 189,000 platelets/mcL. A comprehensive metabolic panel was significant for a creatinine of 1.4 mg/dL and glomerular filtration rate of 32 mL/min/1.73m^2^. Electrophysiology and vascular surgery were consulted. They noted that if they were to proceed with intervention it would include the removal of the pacer wires and vascular stenting with an eventual transition to a leadless pacemaker. However, given the chronic nature of the obstruction, as indicated by the imaging and the patient's symptoms, the patient's contraindications to anticoagulation, age, and comorbidities, they did not feel the patient was a good candidate for the aforementioned procedures. Systemic anticoagulation and antiplatelet therapy after stent placement were relatively contraindicated in this patient who carries a high risk of bleeding complications due to her age, history of hypertension, and history of life-threatening gastrointestinal bleeding. That is why she was not already anticoagulated for stroke prophylaxis in the setting of atrial fibrillation. Treatment options were discussed with the patient and her family, and it was ultimately decided that the patient would not undergo any procedures. The patient had a baseline Clinical Frailty Scale of 6, and she did not feel as if her functional status had been impacted by her onset of symptoms. With a more than 150% greater risk of complications within 30 days after surgery as calculated by the American College of Surgeons National Surgical Quality Improvement Program (NSQIP) Surgical Risk Calculator, the fear was that her quality of life would be diminished by more aggressive therapy [3]. Therefore, the patient was managed conservatively with surveillance and local compressive therapy of the right upper extremity. One year later, the patient still has a Clinical Frailty Scale of 6 and continues to enjoy spending time with her family.

## Discussion

SVC syndrome is a group of signs and symptoms that result from obstruction of the SVC. It is most often caused by malignancy, but non-malignant causes comprise approximately 35-40% of cases. Device-related SVC syndrome is the most common cause of non-malignant SVC syndrome and is seen in approximately 60-65% of cases [1,2]. The incidence of device-related SVC syndrome is rising with the increased use of indwelling catheters, pacemakers, and defibrillators. Thrombotic or embolic complications following transvenous pacemaker implantation have been reported to be 0.6-3.5%, with lead-associated SVC syndrome arising in less than 1% of these cases [4,5]. The pathophysiology is proposed to be repeated trauma and endothelial disruption from the pacemaker leads causing inflammation and resulting fibrin deposition, thrombus formation, and eventually fibrosis with stenosis or obstruction [5]. Risk factors for the development of thromboembolic complications from pacemakers include infection of the leads, implantation of multiple leads, temporary pacemaker implantation prior to permanent pacemaker placement, hormone therapy, and previous venous thrombosis. One study showed that temporary pacemaker implantation or a left ventricular ejection fraction of 40% or less was an independent risk factor for venous stenosis and thrombosis six months after permanent pacemaker insertion [6]. This is particularly significant since cardiac resynchronization therapy and/or defibrillator implantation is potentially indicated in patients with reduced ventricular systolic function. The most common presenting signs and symptoms of SVC syndrome are facial edema (60-100%), non-pulsatile distended neck veins (27-86%), distended chest veins (38-67%), dyspnea and cough (23-70%), and arm edema (14-75%) [1,7]. Symptom severity is related to the acuity of the obstruction. Patients with chronic obstruction may present with mild symptoms or even be asymptomatic. Hemodynamic compromise is rarely seen, and acute mortality is only reported to be 0.3% [8]. The diagnosis is made through history, physical exam, and imaging. Computed tomography of the chest with peripherally administered intravenous contrast provides effective visualization of the SVC and identification of obstruction.

Treatment of SVC syndrome secondary to thrombosis as a complication of pacemaker wires is most often treated endovascularly. Two approaches can be considered: lead removal, stent implantation, and reimplantation of new leads; or angioplasty with balloon dilation of the vein and, in some cases, stent placement as well. Most patients are also treated with a period of systemic anticoagulation. Complications from these endovascular approaches are reported to be 3.6% [5]. The techniques are successful in resolving symptoms in 97.3% of cases with a 30-day mortality of 0.0% [5]. Open surgical techniques can also be considered; this is most often employed for other etiologies of SVC syndrome with mediastinal fibrosis being most common. Complication rates are higher with surgery compared to endovascular interventions, but long-term patency is superior. Both methods require frequent follow-up, and the need for repeat intervention is common.

## Conclusions

SVC syndrome is an uncommon but important potential complication to consider when caring for patients who have indwelling venous devices, particularly central venous catheters and pacemakers. As our patient population ages, we will treat more patients with pacemakers, and early recognition of SVC syndrome as a complication of pacemaker leads will help reduce morbidity associated with the syndrome. Mortality due to SVC syndrome is extremely rare, and therapy is typically aimed at symptom relief or treating the underlying cause, as is the case in malignant SVC syndrome. Therefore, it is reasonable to avoid invasive interventions in patients who are at high risk for perioperative complications and whose symptoms are mild. The patient we presented had minimal symptoms due to the development of good collateral circulation, and due to her age, frailty, and comorbidities, she was at high risk for perioperative complications. The patient’s symptoms were managed with local compressive therapy of the upper extremity. At one year follow-up, the patient's functional status was maintained.
